# Hairless Canaryseed: A Novel Cereal with Health Promoting Potential

**DOI:** 10.3390/nu10091327

**Published:** 2018-09-19

**Authors:** Emily Mason, Lamia L’Hocine, Allaoua Achouri, Salwa Karboune

**Affiliations:** 1 Saint-Hyacinthe Research and Development Centre, Agriculture and Agri-Food Canada, 3600 Casavant Boulevard West, St-Hyacinthe, QC J2S 8E3, Canada; emily.mason2@mail.mcgill.ca (E.M.); allaoua.achouri@Canada.ca (A.A.); 2 Department of Food Science and Agricultural Chemistry, Macdonald Campus, McGill University 21, 111 Lakeshore, Ste Anne de Bellevue, QC H9X 3V9, Canada; salwa.karboune@mcgill.ca

**Keywords:** canaryseed, cereal protein, bioactive peptide, antioxidant, ACE inhibitor, DPP-IV inhibitor, gluten-free, functional food

## Abstract

Glabrous canaryseeds were recently approved for human consumption as a novel cereal grain in Canada and the United States. Previously, canaryseeds were exclusively used as birdseed due to the presence of carcinogenic silica fibers; therefore the nutritional value of the seeds has been seriously overlooked. Two cultivars of glabrous canaryseeds (yellow and brown) were created from the hairy varieties. They are high in protein compared to other cereal grains, and contain high amounts of tryptophan, an amino acid normally lacking in cereals, and are gluten-free. Bioactive peptides of canaryseeds produced by in vitro gastrointestinal digestion have shown antioxidant, antidiabetic, and antihypertensive activity. The seeds contain other constituents with health promoting effects, including unsaturated fatty acids, minerals, and phytochemicals. Anti-nutritional components in the seeds are comparable to other cereal grains. Because of their beneficial health effects, canaryseeds should be regarded as a healthy food and have immense potential as a functional food and ingredient. Further research is required to determine additional bioactive peptide activity and capacity, as well as differences between the yellow and brown cultivars.

## 1. Introduction

Due to the growing global demand for protein, there will be increased need for good sources of high quality plant protein for food uses. Discovering new sources of plant food proteins, besides the conventional ones (ex. wheat, soybean, pulses) provide promising opportunities in terms of environmental sustainability, economic profitability, and nutritional advantages. The consumption of different plant proteins can ensure an adequate supply of essential amino acids for meeting human physiological requirements. Opportunities are endless for using plant proteins as a functional ingredient in formulated food products to increase nutritional quality, as well as to provide desirable health promoting effects. 

In 2015, Health Canada and the Food and Drug Administration (FDA) gave GRAS (Generally Regarded as Safe) status to glabrous canaryseeds (*Phalaris canariensis* L.) and approved them as a novel food product. Previously, the seeds had limited use as birdseed, because they were lined with fine, hair-like silica fibers, that were deemed hazardous to human health [1]. The Crop Development Center at the University of Saskatchewan in Canada developed a new ‘hairless’ or ‘glabrous’ canaryseed from the hairy variety, which is safe for human consumption. Caged and wild birds have consumed hairy canaryseeds for centuries, alone or mixed with other grains, such as millet, sunflower seeds, and flaxseeds [2]. Nonetheless, very little research has been conducted on the seeds, since they had no nutritional value for humans. The new glabrous canaryseed, regarded as a true cereal grain, has tremendous potential in the food industry, due to its unique properties and characteristics. Canaryseed groats contain approximately 61% starch, 20% protein, 8% crude fat and 7% total dietary fiber [3,4]. Compared to other cereal grains in the same family, such as oats, barley, wheat, and rye, they are extremely high in protein. Some studies have shown the potential of hairy canaryseed proteins to produce bioactive peptides with beneficial health effects, such as antioxidant, antihypertensive, and antidiabetic activity [5,6]. Furthermore, unlike wheat, canaryseeds are gluten-free. This review aims to overview the research conducted on canaryseeds to date, particularly the examination of canaryseed proteins and their exceptional health benefits, to ascertain their uniqueness compared to other cereal grains and potential applications in the food industry. 

## 2. Canaryseed Development and Production

**Figure 1:**
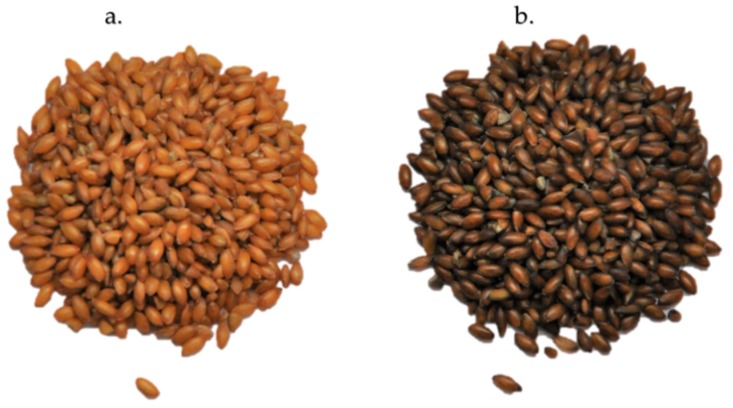
(**a**) Yellow (C09052) and (**b**) brown (CDC Calvi) cultivars of glabrous canaryseeds (*Phalaris canariensis* L.) produced by Hucl, et al. [7], at the Crop Development Center at the University of Saskatchewan.

Hairy canaryseeds, like most grass species, have seeds lined with hair-like silica fibers that were found to be causing lung damage and even esophageal cancer [1]. Hucl, et al. [7], from the University of Saskatchewan’s Crop Development Center (CDC), developed a hairless canaryseed containing no fine hair to decrease skin irritations and potential cancer development by farmers involved in harvesting the crop. The new silica-free or glabrous species was not only safe for individuals manipulating the seeds, but could also be safely consumed and utilized by the food industry as a new cereal grain. Using mutagenesis and breeding techniques, four hairless brown varieties have been created from the original seeds: CDC Maria, CDC Togo, CDC Bastia, and CDC Calvi [8]. In addition, yellow colored cultivars of the glabrous seeds were developed, which are thought to be more aesthetically pleasing for food use as compared to the brown colored cultivar [9] (Figure 1).

Glabrous or hairless canaryseeds are members of the family *Poaceae*, along with other prevalent cereal grains, such as wheat, oat, barley, and rye [10]. The groats (hulled kernels of the grain) have an elliptical shape and measure approximately 4 mm in length and 2 mm in width, comparable to flaxseeds and sesame seeds [4]. The seeds are harvested from canarygrass; a grassy, herbaceous plant that grows optimally in any regions where wheat is cultivated, with growth and production cycles comparable to other winter cereals, such as spring wheat and oat. In addition, very few weeds, diseases, and insects have been reported in canarygrass, which would decrease canaryseed yields [2]. The Western provinces of Canada (Saskatchewan, Manitoba, and Alberta) cultivate the majority of canaryseeds in Canada, which produces over 80% of canaryseed exports worldwide, followed by Argentina and Hungary, mainly to countries with high proportions of caged birds [8]. On average, about 300,000 acres of canaryseed are grown in the province of Saskatchewan every year with yields ranging between 800 to 1400 pounds per acre, representing more than 95 percent of Canadian acreage and production [8], and which is still comprised of only the hairy varieties. The higher yield of the older hairy varieties has limited the uptake by producers of the glabrous varieties. The variety CDC Calvi has the highest yield of the developed glabrous varieties [8]. The approval of glabrous canaryseed varieties for human consumption opens up new opportunities in food applications instead of the sole use as birdseed, which is expected to create more demand for the production of canaryseed.

## 3. Canaryseed Proteins: A Novel Source of Plant Proteins 

### 3.1. Protein Characteristics 

Canaryseeds have been compared extensively with wheat and other cereals in the same family, and one of their distinguishing factors is their higher protein content (Table 1), which ranges between 20–23%, in comparison to 13% for wheat. Canaryseed proteins, along with other cereal proteins, can be separated into four fractions based on their solubility: prolamins, glutelins, globulins and albumins [11]. The prolamin and glutelin fractions, which are principally storage proteins, are more abundant in canaryseeds than wheat, however, the globulin and albumin fractions represent the lowest amount of overall protein [3,4], which is possibly indicative of a reduced amount of anti-nutritional factors, such as enzyme inhibitors [4]. Regardless of the variations in protein fraction proportions, wheat remains unique because of its ability to make dough, due to the exceptional viscoelastic properties of its proteins [11]. Nonetheless, to date, no published data is available on the breadmaking potential of 100% canary flour, although Abdel-Aal, et al. [12] reported that replacement of up to 25% of wheat flour with canaryseed flour in bread had no significant effects on bread quality and loaf volume, except for crumb color. 

A key trait of canaryseeds is their possible lack of gluten-like proteins, which elicit an allergic reaction known as coeliac disease in some sensitive individuals when they consume gluten-containing cereals, such as wheat, barley, and rye [18,19]. Gluten is a complex mixture of proteins called prolamins, which play key roles in conveying dough viscosity/elasticity. Wheat prolamins are termed gliadins and glutenins, barley prolamins are hordeins, and those from rye secalin. A common characteristic of these proteins is the presence of multiple proline and glutamine residues, making them resistant to gastrointestinal digestion and more exposed to deamination by tissue transglutaminase [20]. In a recent study conducted by Boye, et al. [21] to establish the safety of canaryseeds for human consumption from a food allergy perspective, glabrous canaryseeds were analyzed using three separate techniques (enzyme-linked immunosorbent assay (ELISA), mass spectroscopy, and Western blotting) which all yielded negative results for gluten, indicating the cereal is an excellent alternative for individuals with coeliac disease. Although canaryseeds do not contain gluten and may be represented as gluten-free, canaryseeds do however contain a newly reported allergen named granule-bound starch synthase (GBSS), which is present in rice and maize [22], and which cross-reacted with sera from wheat sensitive/allergic individuals [21]. GBSS was simultaneously identified through mass spectroscopy analysis in several cereals (wheat, oat, sorghum, millet, teff, quinoa, buckwheat) [21]. As such, Health Canada has deemed it inappropriate for canaryseed, or food containing canaryseed, to be labelled as “wheat-free”. Health Canada also requires canaryseed and foods containing canaryseed to be labelled with a statement to the effect that the product “may not be suitable for people with wheat allergy”, provided the food does not also contain wheat as an ingredient [10]. 

The amino acid profile of canaryseeds (Table 2) remains unique, due to its high content of tryptophan, an essential amino acid, which is usually lacking in most cereal grains. Abdel-Aal, et al. [4] reported a higher tryptophan content in the Keet cultivar of hairy canaryseed proteins (2.8 g/100 g of protein) as compared to wheat (1.2 g/100 g) and casein (1.0 g/100 g) protein, as well as higher amounts of essential amino acids phenylalanine, leucine, and isoleucine as compared to wheat. Similarly to other cereals, canaryseeds are deficient in essential amino acids lysine, threonine, and methionine, but possess comparable levels to wheat [4]. Glabrous canaryseeds would make an excellent addition to other cereal grain and legume products to ensure consumers meet the recommended dietary intake of essential amino acids. In addition, canaryseeds contain high amounts of glutamic acid. Glutamic acid is the most abundant amino acid in the brain, which plays significant roles in synaptic activity, memory, and learning, also, it was reported that changes in glutamic acid metabolism and regulation in the brain leads to the development of Alzheimer’s disease [23]. Moreover, high content of glutamic acid in the seeds could indicate the presence of high gamma-aminobutyric acid (GABA), a functional compound produced in plants primarily by the decarboxylation of L-glutamic acid, which has several health promoting properties, including reducing blood pressure and blood cholesterol, anticancer, and anti-obesity activity [24]. GABA concentration, however, has not been directly determined in canaryseeds. 

### 3.2. Health Promoting Properties of Canaryseed Proteins

Chronic disease is of major global concern today and includes diseases such as cardiovascular disease, cancer, and diabetes, which are leading causes of death worldwide [27]. A balance between an active lifestyle and good eating habits are critical in the long term to prevent and combat chronic diseases. Beyond their physiological and metabolic effects, dietary proteins are intrinsically associated with health improvement and prevention of nutrition related chronic diseases (ex. cardiovascular diseases, hypertension, cancer, oxidative damage, etc.), and which need to be also considered when assessing protein quality [28]. This is particularly relevant as consumers are increasingly looking to natural food sources to help prevent specific diseases or illnesses. Some parts of world, such as Mexico, have utilized hairy canaryseeds as a traditional folk medicine for treatment of diabetes and hypertension for centuries [5]. However, because of the presence of toxic hairs, the seeds were not consumed directly but soaked in water, drained, dried and then processed to make canaryseed “milk”, which can be safely consumed.

The health benefits associated with drinking canaryseed “milk” were found to be largely related to the bioactive peptides produced during digestion. Bioactive peptides are small, specific and active protein fragments released from food proteins by proteolytic enzymes during protein digestion, which positively affect an individual’s overall health [29,30]. Bioactive peptides have been reported from many food sources, such as fish and crustaceans, dairy products (milk, cheese, yoghurt), eggs, meat, and vegetal sources (grains, legumes, seeds) [31]. Depending on the amino acid composition and sequence, bioactive peptides possess different types of activity, including antioxidant, antimicrobial, antihypertensive, radical scavenging, anti-inflammatory, opioid, immunomodulatory, anticancer, chelation activity, and antidiabetic activity among others [31,32]. In recent years, a lot of research has been focused on the ability of plant proteins from cereals, nuts, and pulses to generate bioactive peptides with measurable health benefits. Thus far, very little research has been conducted on the bioactivity of glabrous canaryseeds. Research on canaryseed proteins and peptide bioactivity has been tested exclusively in vitro to date, with no animal or human subjects, and predominantly using the hairy varieties. Although the nutrient profile between hairless and hairy canaryseeds are very similar, further investigation into hairless canaryseed bioactivity is required and ongoing. 

#### 3.2.1. Antidiabetic Activity 

Dipeptidyl peptidase IV (DPP-IV) enzyme plays a major role in the development of hyperglycemia in individuals with type II diabetes, because it inactivates incretin hormones, thereby increasing blood glucose levels [29]. Incretin-based therapy is a common treatment for type II diabetes, but it remains less effective, because the half-life of the hormone is very short, due to inactivation by DPP-IV enzymes [30]. DPP-IV inhibitors improve the efficiency of incretin-based therapy by inactivating the enzyme and increasing the activity of the incretin hormones. Estrada-Salas, et al. [5] found that peptides produced by in vitro gastrointestinal digestion of canaryseed milk using pepsin, trypsin, and pancreatin, displayed inhibitory activity in a dose dependent manner against DPP-IV enzyme from porcine kidney. In addition, an in vivo and in vitro study have demonstrated an anti-obesity effect of a lipid extract (produced by hexane extraction) of hairless canaryseed [33,34]. The anti-obesity effect of canaryseeds in addition to the inhibitory action of DPP-IV by canaryseed peptides would make this grain an excellent nutritional approach to improve the efficiency of synthetic drugs, since food derived DPP-IV inhibitors lack the potency of synthetic drugs inhibitors [35]. Further characterization of the DPP-IV inhibitor peptides in canaryseeds remains necessary to establish their antidiabetic effects and capacity. 

#### 3.2.2. Antihypertensive Activity 

The angiotensin-I converting enzyme (ACE) increases blood pressure and causes hypertension in inclined individuals. ACE converts the inactive angiotensin-I into angiotensin-II (a very powerful vasoconstrictor) and inactivates bradykinin (a vasodilator), which both lead to the direct increase in blood pressure [5,36]. Synthetic ACE inhibitors are produced as a treatment for hypertension, and although effective, the synthetic inhibitors cause side effects, including coughing, food taste alterations, rashes and reduced efficiency when used in the long term [36]. Food sources of ACE inhibitors are of great interest, since individuals with hypertension can consume them as part of a healthy diet to reduce their high blood pressure [37]. 

Recent research studies revealed that canaryseed bioactive peptides have great potential to lower blood pressure through the inhibition of the ACE enzyme. Estrada-Salas, et al. [5] showed that canaryseed flour proteins digested in vitro using pepsin, trypsin, and pancreatin, exhibited a maximum percent inhibition against the ACE enzyme of 73.5% and an IC_50_ value of 322 μg/mL, which was similar to the IC_50_ value of other peptides from chickpea, pea, soybean, wheat gliadin, and sardine muscle. Undigested canaryseed proteins had significantly lower inhibition activity, meaning the antihypertensive bioactive peptides are produced upon protein digestion [5]. Similarly, Valverde, et al. [6] found that canaryseed flour proteins from the prolamin fraction had the highest inhibition activity against the ACE enzyme, with an IC_50_ value of 217.4 μg/mL, after in vitro digestion with pepsin and pancreatin. They further identified five peptides by mass spectroscopy (LSLGT, TDQPAG, QQLQT, FEPLQLA, and KPQLYQPF) in the digested prolamin fraction that had both ACE and DPP-IV inhibition activity. Additionally, Passos, et al. [38] administered to rats an aqueous extract of canaryseeds (obtained by soaking the seeds in water), which successfully reduced systolic blood pressure in the animals while having no renal or toxicological effects. All these studies demonstrated the potential positive effect of canaryseeds on cardiovascular disease control. 

#### 3.2.3. Antioxidant Activity 

The antioxidant potential of plants has received a great deal of attention, because increased oxidative stress has been identified as a major causative factor in the development and progression of several life threatening diseases, including neurodegenerative and cardiovascular diseases. Free radical species that are generated in the body by various endogenous systems cause extensive damage to body tissues by destroying cell membrane structure, modifying enzyme activity, and changing DNA leading to cancer development [39]. In this regard, bioactive peptides of canaryseeds demonstrated antioxidant activity by reacting with free radical species, thereby preventing tissue damage and decay. Valverde, et al. [6] used two in vitro radical scavenging assays on digested canaryseed protein fractions and found that the prolamins had the overall highest antioxidant activity. Mass spectroscopy analysis of the digested prolamin fraction identified five peptides, of which only one had antioxidant activity (KPQLYQPF). Protein fractions from digested canaryseeds had higher antioxidant activity in general as compared to raw flour, because the seed proteins undergo hydrolysis, increasing their antioxidant activity [6]. 

#### 3.2.4. Other Bioactivities

Only very limited studies have been conducted on other bioactive properties of hairy canaryseed proteins. As an example, acetylcholinesterase inhibitors are currently employed as a form of treatment for individuals with Alzheimer’s disease, because they help maintain levels of acetylcholine in the brain, which is essential for nerve impulses and transmission [40]. Kchaou, et al. [41] found that a methanol extract of a hairy Tunisian canaryseed variety had a percent inhibition against acetylcholinesterase enzyme of 65% at a concentration of 1 mg/mL, which was attributed predominantly to polyphenols and flavonoids in the extract. An antibacterial activity of hairy Tunisian canaryseed extracts, especially against gram-positive bacteria, was also reported by Kchaou, et al. [41]. These bioactivities could possibly be the result of canaryseed peptides, as it was previously demonstrated for hemp seed protein hydrolysates, which exhibited acetylcholinesterase inhibition [40], or for other cereal proteins, such as wheat and barley, for which antibacterial activity was reported [42]. Proteins and peptides from cereal grains and legumes (wheat, barley, amaranth, oat, rye, soybean etc.) are known to have antithrombotic, immunomodulatory, and anticancer activity [42,43,44,45,46,47,48,49,50,51,52]. Bioactivities of Canadian glabrous canaryseed peptides remain largely unknown, but because of the diverse bioactivity reported in similar cereal grains from the same family, it remains highly likely that canaryseed peptides possess additional health promoting properties, which still need to be confirmed. 

### 3.3. Protein Digestibility 

Protein digestibility is an important parameter to consider when assessing protein quality [53]. The health advantages of glabrous canaryseeds depends on their digestibility and bioavailability. Several in vivo studies indicated excellent protein digestibility of canaryseed in animals. Broiler chickens fed hairless canaryseed groats and hulled seeds exhibited similar ileal protein digestibility as other feed components, including corn, wheat, sorghum, and peas [54]. The same study showed high apparent ileal digestibility of amino acids cysteine (86%), phenylalanine (88%), and tryptophan (93%). Furthermore, weight gain between broiler chickens fed with wheat and chickens fed with canaryseeds were similar. 

Later, Classen et al. [55] fed broiler chickens yellow glabrous canaryseeds and glabrous brown seeds and determined the seeds were equivalent in terms of feeding value. Magnuson, et al. [56] found no evidence of toxicity in rats when fed glabrous canaryseeds for a 90 day study and, furthermore, rat diets supplemented with 50% hulled and dehulled glabrous canaryseeds were comparable in terms of growth, hematology, and clinical parameters as rats with diets supplemented with 50% wheat. Thacker [57] showed that crude protein digestibility in pigs increased linearly with increasing proportions of canaryseeds in their diets. Moreover, he found that a pig’s diet containing 25% canaryseeds promoted the highest growth rates in the pigs with a crude protein digestibility of approximately 78%. All these studies indicate that hairless canaryseeds make an excellent addition or supplement to conventional animal feed, as it promotes growth, but also enhances protein digestibility.

For human digestibility of canaryseed proteins, no in vivo study has been reported in the literature despite several in vitro studies that have been carried out to mimic human protein digestibility of canaryseeds under gastrointestinal conditions. Abdel-Aal, et al. [4] used a multienzyme approach with trypsin, chymotrypsin, and peptidase and established an in vitro protein digestibility of 84% in hairy canaryseeds. Interestingly, Rajamohamed, et al. [58] compared the effects of thermal treatment on canaryseed protein digestibility. The in vitro protein digestibility of raw, roasted, and boiled glabrous canaryseed flours was determined by gastric, duodenal, and sequential gastric-duodenal methods. The sequential gastric-duodenal method was most effective at digesting the proteins and, overall, thermal processing enhanced protein digestion. As a cereal, canaryseeds can be used in various forms, such as a whole groat, whole meal, or whole grain flour in several applications, such as a cereal, in pasta, and in baking to make products, such as bread, muffins, and cereal grain bars [10]. Since thermal processing increased protein digestibility, the heating and thermal processing of canaryseeds in the development and production of baked goods will contribute to its improved nutritive value. 

## 4. Other Health Promoting Canaryseed Components 

### 4.1. Starch 

Canaryseeds are comprised of 61% starch, which serves as the main energy store in the plants [59]. Canaryseed starch granules are small and polygonal in shape with reported sizes ranging from 0.5 to 7.5 μm [60,61,62]. X-ray diffraction patterns of the starch exhibit the traits of an A-type starch, characteristic of most cereal grains [61,62]. Starch is comprised of two glucose polymers; linear amylose and branched amylopectin. Abdel-Aal, et al. [62] reported a range of amylose content in hairy canaryseeds of 16.2–19.5% of total starch and Irani, et al. [61] determined an average of 23.6% and 22.5% for a brown and yellow hairless cultivar, respectively, which is typical of most starches [63]. The amylose to amylopectin ratio is indicative of its digestibility because, in general, high amylose starches are harder to digest whereas waxy starches are more readily digested [64]. 

Starches of the yellow and brown cultivars of glabrous canaryseeds have been extensively compared. Overall, their properties appear similar, but some researchers report differences among the two colored cultivars. Irani, et al. [65] observed differences in starch granule shape between a yellow and brown hairless canaryseed variety (CO5041 and CDC Maria, respectively) in dilute solution. The yellow cultivar starch showed both spherical and ellipsoidal structure, whereas the brown cultivar and wheat starch showed only ellipsoidal structure. An investigation of the rheological properties of canaryseed starches revealed C05041 starch was less sensitive to temperature and with increasing concentration, displayed higher thixotrophy and pseudoplastic behavior as compared to CDC Maria starch [66].

Retrogradation, the process of heating starch in the presence of water followed by cooling, results in a critical change in the ordered amylose/amylopectin structure, and hence, in changes to its physiochemical and functional properties. Although starch retrogradation is mostly considered an undesirable phenomenon, such as its involvement in the staling of bread and sensory and quality loss in high starch foods over time, it also plays a nutritionally important role [67]. The retrogradation process can produce resistant starch (also known as resistant starch 3 (RS3)), because the amylose and amylopectin structures become more compact and therefore resistant to enzymatic hydrolysis. Resistant starch is characterized as starch that remains mostly undigested by enzymes in the small intestine, thereby passing into the large intestine where it undergoes fermentation by the colons microflora [68]. There is no rapid release of glucose into the bloodstream and the starch acts like a prebiotic for the gut microflora. Canaryseed starch demonstrated greater rates of hydrolysis in the presence of pancreatic α-amylase as compared to wheat starch, which could be due to its small granule size and relatively low amylose composition [62]. Nonetheless, canaryseed starch also had a higher tendency for retrogradation, potentially forming RS3, a nutritionally valuable starch. Resistant starches promote probiotic bacteria, lower the glycemic index of foods, have hypocholesterolemic effects, reduce gallstone formation, improve mineral absorption, have high satiety, and aid in weight management [69]. 

Overall, canaryseed starch does possess unique characteristics as compared to wheat starch. Its properties in dilute solution are similar to that of wheat and demonstrate a potential use as a thickener or stabilizer in food products [61]. Canaryseed starches, although easily digestible, have a higher tendency to retrograde into RS3, which could make them more available for digestion by the colons microflora [61,62]. This functionality, however, would need to be further investigated.

### 4.2. Fiber 

Besides starch and protein, fiber represents a minor component of the total composition of canaryseeds. Canaryseeds consist of approximately 7% dietary fiber, considerably lower compared to other cereal grains, especially wheat, which contains double the amounts on average [4,12,70]. The bran portion of the grain contains more dietary fiber than the whole grain and white flour portions in both canaryseeds and wheat [12]. Several purification steps are usually required to obtain a high purity fiber, due to high contamination with starch and protein. The extraction order also plays a role on fiber extraction purity, since the removal of starch and protein prior to fiber in an ethanol, alkaline, and water wet milling extraction technique results in a higher fiber purity [3]. Overall, canaryseeds still remain a poor source of dietary fiber compared to other grains from the same cereal family. 

### 4.3. Lipids

Similarly to fiber, lipids are minor components of the seeds as compared to starch and protein. To extract oil from canaryseeds, ethanol has proved a very suitable solvent. Abdel-Aal, et al. [3] reported a crude oil content of 8.3% with an extraction efficiency of 75% when the ethanol extraction step was repeated three times. Oil from canaryseed would be produced primarily as a byproduct, since its removal is necessary to obtain purified starch and protein fractions from the seeds. 

The crude fat content in glabrous canaryseed is high as compared to other cereal grains and the fatty acids are largely unsaturated (Table 3). Canaryseeds lipids consist of 54% linoleic, 29% oleic, 11% palmitic, 2.4% linolenic, and 1% stearic acids [8]. In comparison, wheat grain lipids consist of 62% linoleic, 16% oleic, 17% palmitic, 4% linolenic, and 1% stearic acids [4]. Diets high in saturated fatty acids have been correlated with increased incidence of chronic heart disease, whereas diets higher in monounsaturated fatty acids (oleic acid) and especially polyunsaturated fatty acids (linoleic acid, linolenic acid) promote cardiovascular health, neurological function, and improved immune response [71]. Canaryseeds contain high amounts of unsaturated fatty acids, which is advantageous for a healthy diet, but could make them prone to oxidation and rancidity. However, the presence of certain antioxidants in canaryseed oil, such as caffeic acid esters, could potentially reduce these detrimental effects [72]. Furthermore, Ben Salah, et al. [73] reported health promoting activity in canaryseed oil, produced from a hairy Tunisian canaryseed variety, which demonstrated antioxidant, antibacterial, and antiacetylcholinesterase activity, which was largely attributed to the high total polyphenol content in the oil. 

### 4.4. Minerals 

In terms of nutrients, glabrous canaryseeds contain several essential minerals and are higher in phosphorous, magnesium, and manganese compared to wheat, oat, barley, and millet, nonetheless, although comparable to levels present in wheat, canaryseeds contain less iron and calcium as other cereal grains (Table 4). Canaryseeds contain higher amounts of vitamin B1 (thiamine) as compared to wheat and an equivalent amount of vitamin B2 (riboflavin), but are poor in niacin [12]. 

### 4.5. Phytochemicals 

Phytochemicals, including polyphenols, terpenoids, and alkaloids, are naturally occurring chemicals produced by plants and, when consumed, promote positive overall health. Research indicates that glabrous canaryseeds are a good source of different types of phytochemicals. Ferulic acid is the most abundant phenolic acid in canaryseeds [79,80,81]. Ferulic acid displays a broad range of health promoting effects, including anti-inflammatory, antidiabetic, antiaging, neuroprotective, radioprotective, and hepatoprotective activity, mainly due to its strong antioxidant activity [82]. Li et al. [81] compared the total phenolic and flavonoid content in nineteen different samples of brown and yellow varieties of canaryseed groats. They found the yellow and brown colored seeds had the same flavonoid profiles and that ferulic acid was the dominating phenolic acid, followed by caffeic and coumaric acid, but unlike their flavonoid profiles, brown cultivars had higher amounts of ferulic and caffeic acid relative to the yellow cultivars [81]. *O*-pentosyl isovitexin, identified as the major flavonoid in canaryseeds, displays diversified activity including anti-hypotensive, anti-inflammatory, antimicrobial, antiplatelet, and antioxidant [81]. 

Carotenoids are another class of phytochemicals that, when ingested, perform a number of beneficial biological functions, including antioxidant activity, immune response improvement, suppression of reactive oxygen species, and lowering the risk of cardiovascular disease [83]. Cereals in general possess only small amounts of carotenoids as compared to fruits and vegetables, nonetheless, the pigment remains present and concentrated mostly in the bran fraction. The major carotenoids present in cereals are xanthophylls like lutein, zeaxanthin, and β-cryptoxanthin with only small amounts of carotenes [83]. Li and Beta [84] evaluated the total carotenoid content in brown and yellow glabrous canaryseed cultivars and determined lutein, zeaxanthin, and β-carotene were the three major carotenoids present. Surprisingly, β-carotene was present in the largest quantities in all canaryseed varieties and far outweighed the β-carotene content of other crops, including wheat, rice, barley, and corn [84]. The carotenoid content of the brown and yellow canaryseed cultivars were relatively similar, in contrast, canaryseed flour was significantly higher in total carotenoid content (11.28 mg/kg) compared to the whole meal (9.27 mg/kg), and bran (8.32 mg/kg) fractions [84]. The results indicate canaryseed flour is a good source of carotenoids. However, carotenoids are highly sensitive molecules and changes in carotenoid stability during storage and processing still need to be addressed.

### 4.6. Anti-Nutritional Components 

Like all cereal grains, canaryseeds contain certain anti-nutritional factors, including enzyme inhibitors, amylase inhibitors, phytate, and heavy metals. Enzyme inhibitors play important roles in living plants by preventing proteins and carbohydrates from degradation during growth and protection against threats by animals, insects and some microorganisms [11]. Trypsin inhibitor is a type of enzyme inhibitor present in raw cereals and legumes and, upon consumption, could lead to reduced protein and nutrient digestibility and even cause growth inhibition [79]. Likewise, amylase inhibitors form aggregates with amylase, resulting in a reduction of starch digestion when consumed [85]. 

Phytate can also be considered as both nutritional and anti-nutritional component in cereals. Phytate has chelating properties and could reduce the availability of some essential minerals, like calcium, iron, and zinc, thereby decreasing their absorption in the small intestine, but on the other hand, exhibits antioxidant activity showing positive effects in cancer treatment, hypercholesterolemia, hypercalcuria, and kidney stones [79]. Similarly, heavy metals present in raw cereals are essential to human health and provide beneficial effects (acting as cofactors to essential enzymes and aiding in the production of amines and amino acids). 

Abdel-Aal, et al. [79] evaluated the trypsin inhibitor, amylase inhibitor, phytate and heavy metal content in the bran, wholegrain flour, and white flour of hairy canaryseeds, hairless canaryseeds, and wheat. All hairless canaryseed fractions contained significantly more phytate than wheat (28–41%), but no significant difference in trypsin inhibitor content compared to wheat. Canaryseed amylase inhibitor content was higher in the white flour fraction, but lower in the bran fraction as compared to wheat. 

With regards to heavy metals, the hairless canaryseed variety CDC Maria contained higher amounts of the essential heavy metals zinc (44.8 mg/kg), nickel (2.27 mg/kg), and copper (38.0 mg/kg) as compared to the wheat control (32.24 mg/kg, 0.34 mg/kg, and 24.4 mg/kg for zinc, nickel, and copper respectively), however, the molybdenum content was higher in wheat (0.64 mg/kg) as compared to CDC Maria (0.51 mg/kg) [79]. There was no significant difference in neutral metal content (antimony, cobalt, selenium, tellurium, tungsten), and toxic metal content (arsenic, cadmium, lead, mercury), between CDC Maria and the wheat control, and all toxic metals were present in acceptable levels to human health for both grains. 

In summary, the anti-nutritional components of wheat and glabrous canaryseeds are very similar and the anti-nutrients are present in low enough quantities that they do not outweigh their positive health benefits. To date, no studies compare the anti-nutritional components of multiple varieties of glabrous yellow and brown seeds. Li, et al. [81] reported a difference in phenolic acid content between brown and yellow canaryseed cultivars and a similar trend could exist in terms of their anti-nutritional content. 

## 5. Potential as a Functional Food and Alternative to Major Allergens

Functional foods are a growing trend among consumers today, because consumers not only eat food to satisfy their hunger, but they eat specific foods to maintain or improve their overall health [86]. Although there is no official definition of a functional food, the general idea is their consumption provides exceptional nutritional health benefits above and beyond basic nutrition. Some food products, designated as “superfoods”, offer more than one health promoting property and recent superfood trends among consumers include oats, hemp seeds, almonds, kale, acai berries, blueberries, and green tea among others [87,88,89]. Oats contain large proportions of beta-glucan, a type of water soluble fiber present in the grain that possess several health promoting effects, such as reducing cholesterol and lowering postprandial glucose and insulin levels in the blood, which is especially beneficial for individuals with type II diabetes [90]. Likewise, canaryseeds demonstrate exceptional nutritional qualities, including their antioxidant, antidiabetic, antihypertensive, and even anti-obesity activity. Furthermore, their phytochemical content (phenolic acids, carotenoids, and flavonoids) and relatively low abundance of anti-nutritional factors contribute to their nutritional qualities. The grains themselves could be used as a functional ingredient in food products (such as granola bars, bread, pasta, and cereals) to improve their nutritional value. In addition, canaryseeds are gluten-free. Using canaryseed to replace wheat or gluten-containing cereals will create more options for gluten-sensitive individuals and also produces new opportunities to develop gluten-free products. Moreover, because of their size and shape, canaryseeds offer the possibility to replace sesame seeds in products, such as baked goods, snack foods, and toppings, creating new products for individuals with allergies to sesame seeds. 

## 6. Conclusions

Glabrous canaryseed, technically an ancient grain, is an excellent new source of plant based protein. Confirmation of the broad spectra of its potential bioactivities and health benefits would make this cereal an excellent nutritional and therapeutic aid to help combat non-communicable diseases, including cancer, diabetes, and heart disease. Due to a lack of knowledge, and because the seed is “new”, this unique cereal is currently underutilized by consumers and the industry. However, growing trends among consumers, including the consumption of functional foods and gluten-free products, have created high demands in the food industry that can be supported with the use of glabrous canaryseeds.

## Figures and Tables

**Table 1:** Protein comparison between canaryseed and other cereals.

Cereal Variety	% Protein (Dry Basis)	Reference
**Canaryseed**	20–23%	[4,12]
**Wheat**	13%	[13]
**Oat**	10–13%	[14]
**Barley**	13–16%	[15]
**Rye**	11–16%	[16]
**Millet**	8.5–15%	[17]

**Table 2:** Amino acid comparison between canaryseeds and other cereals.

Amino Acid	Canaryseed (g/100 g Protein)	Wheat (g/100 g Protein)	Oat (g/16 g N or g/100 g Protein)	Barley (g/100 g Protein)	Millet (g/100 g Protein)
**Histidine**	1.6	2.1	1.74	2.4	2.4
**Isoleucine**	3.9	2.8	2.32	3.5	4.4
**leucine**	7.6	5.3	5.26	7.7	11.5
**lysine**	2.6	1.9	2.73	3.9	2.8
**Methionine**	1.9	1.4	2.5	2.1	2.3
**Phenylalanine**	6.5	5.4	5.3	5.7	5.6
**Threonine**	2.7	2.8	2.46	3.9	4.2
**Tryptophan**	2.8	1.2	1.15	N/A	N/A
**Valine**	4.8	3.8	3.2	5.4	6.0
**Alanine**	4.5	3	3.59	4.4	8.8
**Arginine**	6.4	5.1	5.79	4.6	3.9
**Aspartic acid**	4.4	4.4	7.37	6.3	8.7
**Cystine**	2.5	2.3	2.74	1.4	1.2
**Glutamic acid**	26	33	19.12	28.1	22
**Glycine**	3.1	3.8	3.81	4.7	3.2
**Proline**	6.2	8.6	4.54	12.7	6.8
**Serine**	4.5	4.3	3.86	4.9	5.3
**Tyrosine**	3.6	3.5	1.82	2.8	2.4
**Reference**	[8]	[4]	[14,25]	[26]	[26]

N/A = not available.

**Table 3:** Crude fat and lipid composition of canaryseed and other cereal grains.

	Canaryseed	Wheat	Oat	Barley	Millet
**Crude Fat (% dry basis)**	6.7	4.4	4.79	3.4	4.7
Reference	[8]	[4]	[14]	[74]	[74]
**FA (% total lipids)**					
**Palmitic (C16)**	11.38	16.6	19.2	23.0	7.42
**Stearic (C18)**	1.22	0.8	1.46	1.12	6.84
**Oleic (C18:1)**	29.1	16.2	30.8	11.4	16.11
**Linoleic (C18:2)**	53.39	62.1	46.4	58.8	66.68
**Linolenic (C18:3)**	2.42	4.0	2.13	7.78	2.48
Reference	[8]	[4]	[75]	[75]	[76]

**Table 4:** Nutrient comparison between glabrous canaryseeds and other cereal grains.

Mineral	Canaryseed (mg/100 g)	Wheat Grain (mg/100 g)	Oat Grain (mg/100 g)	Barley (mg/100 g)	Millet (mg/100 g)
**Phosphorous**	640	430	340	457	288
**Magnesium**	200	155	140	197	149
**Manganese**	6.3	5.9	5.1	0.92	0.81
**Iron**	6.5	4.2	4.5	12.8	20
**Zinc**	3.9	2.5	3.5	7.4	6.6
**Calcium**	40	20	62	73.6	51
**Potassium**	385	355	420	457	280
**Reference**	[12]	[12]	[77]	[78]	[78]
